# Associations between *ADIPOQ* rs2241766 SNP and breast cancer risk: a systematic review and a meta-analysis

**DOI:** 10.1186/s41021-021-00221-2

**Published:** 2021-11-06

**Authors:** Xue Hu, Chunguo Cui, Tong Sun, Wan Wang

**Affiliations:** Department of Breast Surgery, China-Japan Union Hospital of Jilin University, 126 Xiantai Blvd, 130033 Changchun, China

**Keywords:** Breast, Cancer, Genetics, Meta-analysis, Polymorphism

## Abstract

**Purpose:**

We aimed to conduct a meta-analysis to accurately evaluate the potential association between *ADIPOQ* rs2241766 gene SNP and breast cancer risk.

**Methods:**

A systematic literature search on Cochrane Library, PubMed, Embase, Web of Science and China National Knowledge Infrastructure (CNKI) identified 8 articles with 1692 cases and 1890 controls. Strength of association was evaluated by pooled odds ratio (OR), 95 % confidence interval (CI) and p value. Funnel plots and Begger’s regression test were applied for testing the publication bias. Statistical analysis of all data was performed by Stata 12.0.

**Results:**

The meta-analysis results indicated that the *ADIPOQ* rs2241766 gene polymorphism did not significantly associated with the risk of breast cancer for these genetic models (TT vs. TG + GG: OR = 1.20, 95 % CI = 0.77–1.89, *p*=0.417; TT + TG vs. GG: OR = 1.05, 95 % CI = 0.71–1.56, *p*=0.805; T vs. G: OR =1.17, 95 % CI = 0.79–1.74, *p*=0.437).

**Conclusions:**

This study indicated that no significant relationship between the *ADIPOQ* rs2241766 SNP and breast cancer. Further large-scale and well-designed studies will be indispensable to confirm our result.

## Introduction

Breast cancer was the most common malignant tumor in women, which was the second most common malignant tumor worldwide [1]. According to data from the World Health Organization and the International Agency for Research on Cancer in 2012, a total of 1.67 million breast cancer patients were confirmed worldwide. This number accounted for 11.8 % of all tumors [2]. So far, the pathogenesis of breast cancer remains unclear.

Recent studies had shown that adiponectin (*ADIPOQ*) was inversely related to breast cancer and other tumors [3–6]. *ADIPOQ* gene was found on the 3q27 chromosomal expressed by adipose tissue and had more than 620 variants [7, 8]. *ADIPOQ* gene polymorphism was closely related to cancer risk by influencing plasma level of *ADIPOQ* [9]. To date, previous studies on the relationship between the polymorphism of *ADIPOQ* rs2241766 gene and breast cancer susceptibility were limited and rather contradictory [10–17]. For lack of powerful evidence to provide a reliable conclusion in a single study, we conducted a comprehensive meta-analysis to assess the strength relationship between *ADIPOQ* rs2241766 gene polymorphism and breast cancer risk, which would have much greater possibility to reach reasonably reliable conclusions.

## Materials and methods

### Publication search

We systematically searched on database of Cochrane Library, Pubmed, Embase, Web of Science and China National Knowledge Infrastructure, up to October 31, 2019, using the following terms: (“*ADIPOQ*” OR “adiponectin receptor” OR “rs2241766”) AND (“variant” OR “polymorphism” OR “mutation”) AND (“breast cancer” OR “breast tumor”). Two investigators manually checked the reference of retrieved articles and extracted the publications independently. In addition, only English and Chinese articles were included.

### Selection criteria

All selected studies complied with the inclusion criteria: (1) full text can be found; (2) case-control studies focused on relationship between the *ADIPOQ* rs2241766 polymorphism and risk of breast cancer; (3) *ADIPOQ* rs2241766 genotype was obtained. Main exclusion criteria as followed: (1) repeat of other articles; (2) not case–control studies; (3) unpublished studies, conference articles, meta-analysis and systematic evaluations; (4) pedigree studies. Consulting the Preferred Reporting Project (PRISMA) Guide for Systematic Evaluation and Meta-Analysis[18], by screening all retrieved literatures, we constructed an information flow diagram about the final eligible data.

### Data extraction

Two investigators extracted data independently according to the selection criteria. The following items were collected: first author, country, publication year, amount of cases and controls, Hardy-Weinberg equilibrium, control group source and the availability of *ADIPOQ* rs2241766 genotype. Only the article with maximum sample size was selected while same data appearing in multiple publications. To insure the data accuracy, a third investigator reviewed the final results. Discussions were executed to solve disagreements.

### Study quality assessment

Two researchers performed independent quality assessment for each eligible article according to 9-point Newcastle-Ottawa Scale (NOS) applied to quality evaluation of observational studies [19]. Different results from two evaluators were solved by the third assessor. Assessment score principally included these aspects: (1) case and control selection (4 point); (2) Confounding factor quality corrected in cases and controls (2 point); (3) exposure ascertainment (3 point). The total scores ranged from 0 to 9, and scores above 6 indicate high quality.

### Statistical analysis

Odds ratio (OR) and 95 % confidence interval (CI) were calculated to estimate the relationship between *ADIPOQ* rs2241766 gene SNP and the risk of breast cancer. The Chi-square based Q-test and I-squared test was used to analyze the heterogeneity (*P*<0.10 suggested Heterogeneity) [20, 21]. The pooled OR was estimated by fixed effect model (Mantel–Haenszel) when no heterogeneity existed. Otherwise, the pooled OR was estimated by random effect model (DerSimonian and Laird) [22, 23]. In controls, Chi-square test was used to examine Hardy–Weinberg equilibrium (HWE). In order to estimate the influence of the pooled ORs caused by individual data set, we performed sensitivity analysis for each comparison models respectively. The publication bias was tested by Funnel plot and Begg linear regression [24, 25]. Stata 12.0 was used to perform all analysis (Stata Corp, College Station, United States).

## Results

### Characteristic of studies

**Fig. 1:**
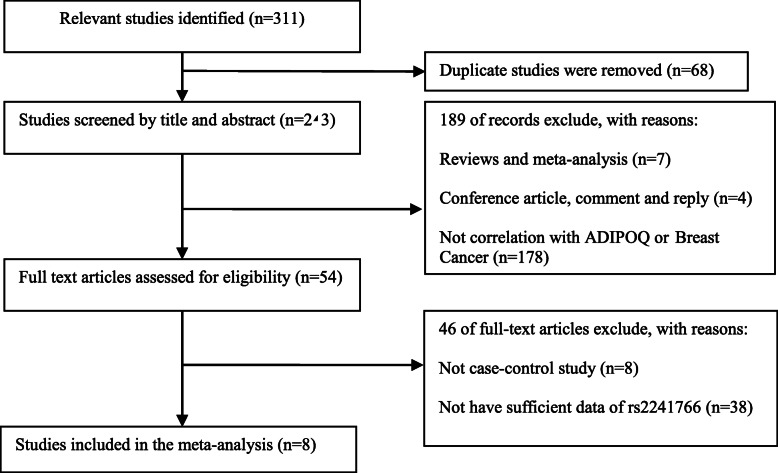
The flow sheet of identification of eligible studies

Flow diagram for the retrieve strategy was demonstrated in Fig. 1. 311 publications were identified initially. 68 duplicate publications were excluded by verified and deleted, while 243 publications entered our study. 54 publications were reviewed for full-text review by reading title and abstract. Ultimately, 8 studies with 1692 breast cancer patients and 1890 controls were screened out for the final meta-analysis which published between 2008 and 2019. The gene distributions in control groups were all consistent with HWE. In addition, all studies were high quality because of the NOS scores ranging from 7 to 8. The relevant feature information was presented in Table 1.

**Table 1:** Studies and data included in this meta-analysis

Author	year	country	Ethnicity	Source of control	Sample size		case					control					NOS score	HWE
					case	control	TT	TG	GG	T	G	TT	TG	GG	T	G		
Macias-Gomez NM et al.	2019	Mexico	Non-Asian	HB	64	167	55	9	0	119	9	115	46	6	276	58	8	0.603
Pasha HF et al.	2019	Egypt	Non-Asian	HB	120	120	76	35	9	187	53	105	13	2	223	17	7	0.053
Kaklamani VG et al.	2013	America	Non-Asian	HB	366	366	330	34	2	694	38	330	35	1	695	37	8	0.944
Kaklamani VG et al.	2008	America	Non-Asian	HB	712	801	524	167	21	1215	209	520	252	29	1292	310	7	0.822
Erbay B et al.	2016	Turkey	Non-Asian	PB	97	101	60	36	1	156	38	68	31	2	167	35	7	0.473
Al Khaldi RM et al.	2011	Kuwait	Asian	PB	60	68	34	26	0	94	26	50	18	0	118	18	7	0.208
Cui HX et al.	2009	China	Asian	PB	68	62	31	28	9	90	46	33	24	5	90	34	8	0.829
Khandouzi M et al.	2016	India	Asian	HB	205	205	132	64	9	328	82	146	50	9	342	68	8	0.090

*HB* Hospital-based, *PB* population-based

### Meta-analysis results

**Fig. 2:**
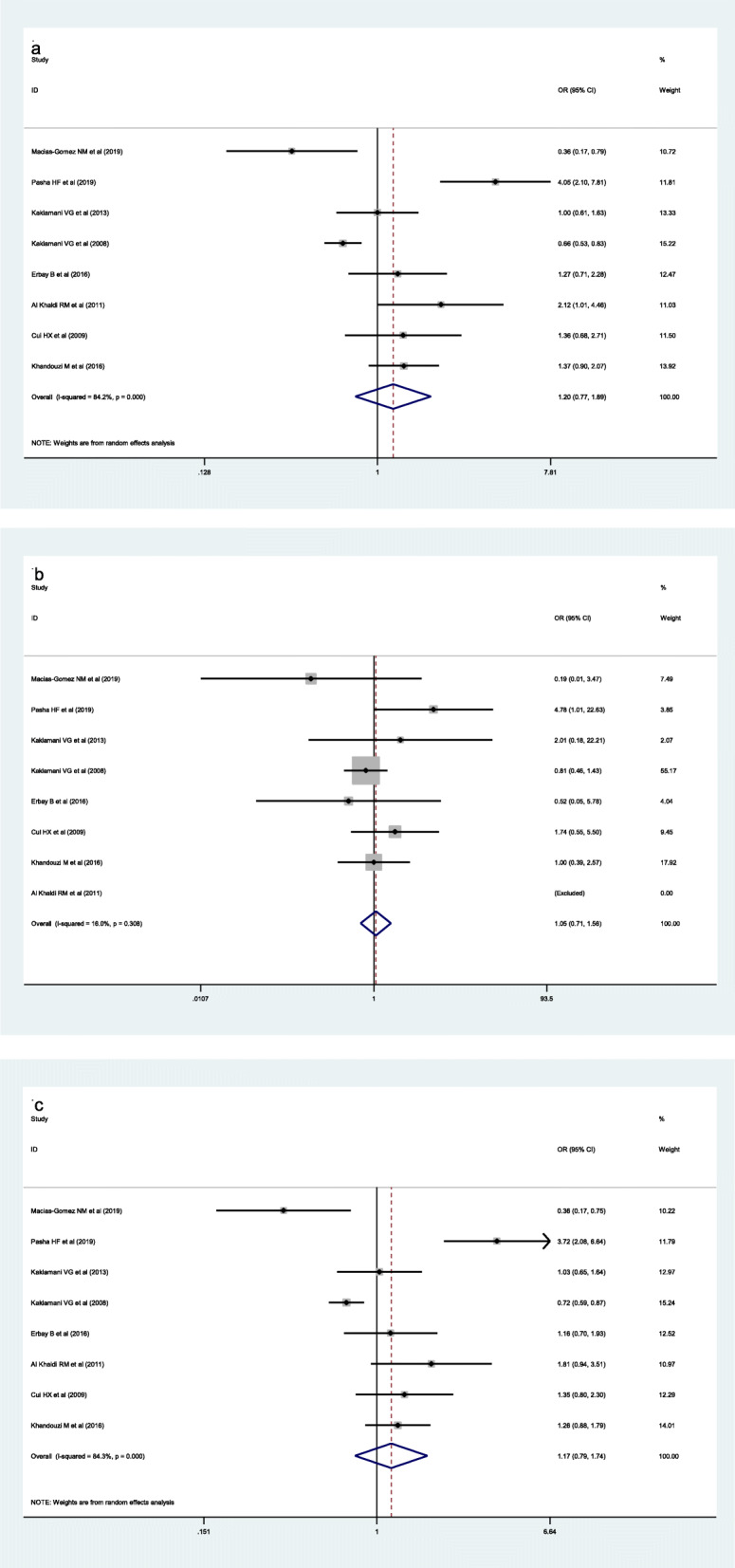
Forest plots of the *ADIPOQ* rs2241766 polymorphism under different genetic models. **A** is the model of TT vs. TG + GG; **B** is the model of TT +TG vs. GG; **C** is the model of T VS G

**Fig. 3:**
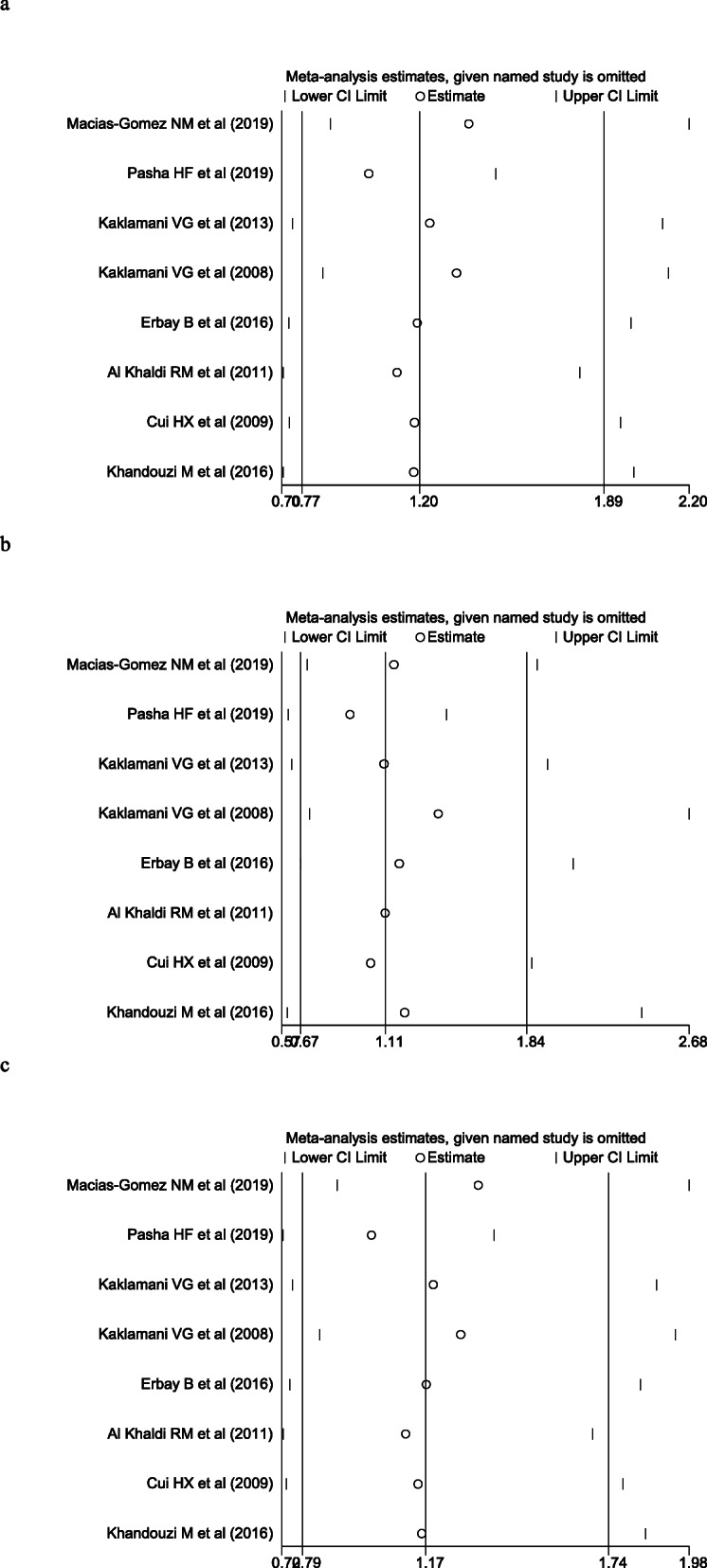
Sensitivity analysis examining the association between the *ADIPOQ* rs2241766 polymorphism and risk of breast cancer under these models (TT vs. TG + GG, TT +TG vs. GG, T vs. G)

**Fig. 4:**
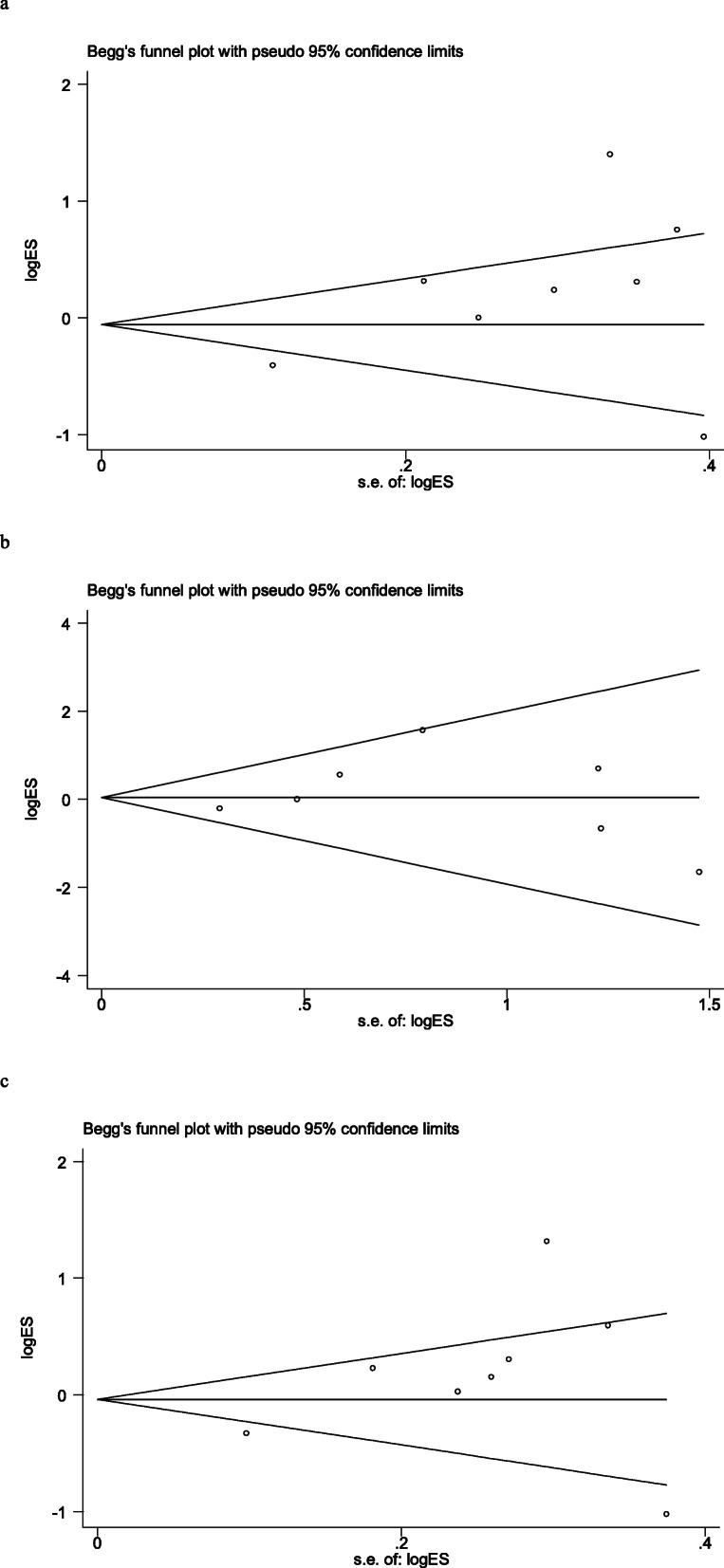
Begg’s funnel plot for publication bias analysis. **A** is the model of TT vs. TG + GG; **B** is the model of TT +TG vs. GG; **C** is the model of T vs. G

Chi-square was used to determine the heterogeneity of the three genetic models. The heterogeneityre was significant in these models according to Table 2 (TT vs. TG + GG (recessive model): *p*<0.001, *I*^2^ = 84.2 %; T vs. G (allele model): *p*<0.001, *I*^2^= 84.3 %). However, the heterogeneity was not considered significant in these models (TT + TG vs. GG (dominant model): *p*=0.308, *I*^2^ = 16.0 %) Fig. 2. So the recessive model and allele models were analyzed by random effect model. The fixed was applied to analyze the dominant model. There was no significant association between *ADIPOQ* rs2241766 polymorphism and breast cancer risk under the model of TT vs. TG + GG (OR = 1.20, 95 % CI = 0.77–1.89, *p*=0.417), TT + TG vs. GG (OR =1.05, 95 % CI = 0.71–1.56, *p*=0.805), C vs. T (OR =1.17, 95 % CI = 0.79–1.74, *p*=0.437). We also performed a subgroup analysis according to ethnicity, source of control, case size and control size. We find the statistically significant association for the dominant model which observed in Asian population (OR=1.48, 95 %CI=1.08-2.05), control from population (OR=1.49, 95 %CI=1.01-2.18) and studies with less or more than 100 controls (OR=1.67, 95 %CI=1.01-2.77), and less or more than 100 cases. The pooled OR and 95 % CI of the relationship between *ADIPOQ* rs2241766 polymorphism and breast cancer were in Table 1. A sensitivity analysis was conducted to reflect the effect of each independent study on the whole studies. As shown in Fig. 3, none of the study affected the overall results of. Begg’s funnel plot was used to assess the publication bias. The results showed that there was no publication bias reflected in three genetic models (TT vs. TG + GG: *p*=0.458; TT + TG vs. GG: *p*=0.881; T vs. G: *p*=0.322) (Fig. 4).

**Table 2:** Pooled ORs and 95 % CIs of the association between *ADIPOQ* rs2241766 polymorphism and breast cancer

Total and subgroups	Studies	TT vs. TG + GG			TT +TG vs. GG			T vs. G		
		OR (95 %CI)	*P* values for OR	*I*^*2*^	OR (95 %CI)	*P* values for OR	*I*^*2*^	OR (95 %CI)	*P* values for OR	*I*^*2*^
Total	8	1.2 (0.77-1.89)	0.417	84.20 % ^*^	1.05 (0.71-1.56)	0.805	16.00 %	1.17 (0.79-1.74)	0.437	84.30 % ^*^
Ethnicity										
Asian	3	1.48 (1.08-2.05)	0.016	0.00 %	1.26 (0.61-2.59)	0.551	0.00 %	1.36 (1.04-1.78)	0.024	0.00 %
Non-Asian	5	0.83 (0.70-0.99)	0.040	88.00 % ^*^	0.97 (0.61-1.56)	0.913	36.10 %	1.04 (0.57-1.87)	0.907	88.70 % ^*^
Source of control										
HB	5	1.06 (0.58-1.95)	0.085	89.10 % ^*^	1.00 (0.65-1.54)	0.997	33.30 %	1.06 (0.60-1.84)	0.849	89.50 % ^*^
PB	3	1.49 (1.01-2.18)	0.041	0.00 %	1.37 (0.50-3.77)	0.539	0.00 %	1.36 (0.99-1.88)	0.056	0.00 %
Case size										
<100	4	1.08 (0.78-1.50)	0.655	74.00 % ^*^	0.95 (0.39-2.31)	0.912	19.10 %	1.04 (0.57-1.88)	0.907	74.70 %
>100	4	0.92 (0.77-1.09)	0.329	90.50 % ^*^	1.08 (0.69-1.68)	0.740	37.30 %	1.31 (0.72-2.37)	0.378	90.60 % ^*^
Control size										
<100	3	1.67 (1.01-2.77)	0.046	0.00 % ^*^	0.98 (0.64-1.49)	0.347	20.10 %	1.52 (1.00-2.30)	0.047	0.00 %
>100	5	0.90 (0.77-1.05)	0.183	86.90 % ^*^	1.74 (0.55-5.50)	0.922	0.00 %	1.07 (0.67-1.72)	0.770	87.20 % ^*^

*HB* Hospital-based, *PB* population-based, *ORs* odds ratios, *CIs* confidence interval; * *p* values for *I*^*2*^ less than 0.05

## Discussion

In recent years, several case–control studies have shown an association between the importance of *ADIPOQ* rs2241766 gene SNP and breast cancer risk, however, to date, it is still inconclusion. The first study had been reported *ADIPOQ* rs2241766 gene polymorphisms in relation to breast cancer risk in 2008 [10], in this study, the association between the high expression of rs2241766 G allele (GT and GG genotype) and low risk of breast cancer was detected. In 2019, Macias-Gomez et al. [16] found that the G allele of *ADIPOQ* rs2241766 polymorphisms and the TG+GG genotype had protective effects on the occurrence of breast cancer. Series of studies supported the conclusion while others objected. Pasha et al. [17] detected that the *ADIPOQ* rs2241766 SNP might cause breast cancer (OR=6.2, 95 %CI=1.3–29.6). Al Khaldi et al. [12] found that individuals with GG genotypes showed an increased risk of breast cancer (OR =2.1, 95 %CI=1.1–4.1). Others studies suggested that there was no association between *ADIPOQ* rs2241766 gene SNP and increased risk of breast cancer [11, 13–15].

In the recessive model (OR = 1.20, 95 %CI = 0.77–1.89), dominant model (OR = 1.05, 95 %CI = 0.71–1.56) and allele model (OR = 1.17, 95 %CI = 0.79–1.74), the *ADIPOQ* rs2241766 polymorphism was not significantly associated with breast cancer risk. Considerable heterogeneity was discovered among these studies. We also conducted a subgroup analysis according to racial classification, case size, control size and source of control. In dominant genetic model, the significant correlation was not found in stratification analysis by racial classification, case size, control size and source of control. But in Asians, the statistically significant association for the dominant model was observed (OR=1.48, 95 %CI=1.08-2.05), control from population (OR=1.49, 95 %CI=1.01-2.18) and studies with less than 100 controls (OR=1.67, 95 %CI=1.01-2.77). Moreover, the significant associations were observed in allele genetic model in stratified analysis among Asians (OR=1.36, 95 %CI=1.04-1.78) and studies with less than 100 controls (OR=1.52, 95 %CI=1.00-2.30). Because of the limited samples and finite studies [11, 12], it would be immature to conclude that the *ADIPOQ* rs2241766 SNP has no relationship with breast cancer. New further discoveries will appear and the information will be constantly renovated. There should be plenty of research data to support more objective results.

The detailed mechanism of the association between ADIPOQ rs2241766 and breast cancer is unclear. Previous epidemiological studies [26, 27] have confirmed a significant association between obesity and some adipokines and breast cancer risk. Logically, adiponectin may play a role in the development of breast cancer. Uncontrolled cellular proliferation is a hallmark of tumorigenesis. Adiponectin is an important regulator of cell proliferation and apoptosis. Adiponectin has been shown to significantly suppress the proliferation of MDAMB-231 cells by arresting the cells at G0/G1 phase and inducing apoptosis [28]. Furthermore, adiponectin also significantly inhibits cell proliferation induced by leptin, oxidized, low-density lipoprotein, platelet-derived growth factor BB, basic fibroblast growth factor (bFGF), and heparinbinding epidermal growth factor-like growth factor [28].

Several limitations in the study should be considered. First, only 8 studies were selected into our meta-analysis. The sample size and amount of studies were comparatively small, which affected the reliability of the results. Second, the case size and control size of some studies were relatively small to confirm the risk of *ADIPOQ* rs2241766 SNP. Finally, in all genetic models, the heterogeneity of the recessive model and allele model may have a significant impact on the result of meta-analysis.

## Conclusions

In conclusion, we find the significant associations in the recessive model and allele genetic model in stratification analysis. The risk of *ADIPOQ* rs2241766 SNP could not be confirmed due to the relatively limited sample and small amount of study. Scholars had conducted extensive research on the susceptibility factors of breast cancer, which believed that the breast cancer occurrence was influenced by multiple factors [29–33], especially genetic factors and life-style. Therefore, the influence of confounding risk factors will be eliminated by further studies, such as age, BMI and environmental factors.

## Data Availability

Not applicable.

## Abbreviations

CNKI: China National Knowledge Infrastructure
ADIPOQ: adiponectin
IARC: International Agency for Research on Cancer
WHO: World Health Organization
NOS: Newcastle-Ottawa Scale
OR: odds ratio
CI: confidence interval
